# Evaluation of the necessity of Pulmonary Ligament Lymph Node Dissection for Upper Lobe Stage IB NSCLC: A Propensity Score-matched Study

**DOI:** 10.7150/jca.76108

**Published:** 2022-09-06

**Authors:** Feng Wang, Xiangyang Yu, Yi Han, Lanjun Zhang, Shuku Liu

**Affiliations:** 1 Department of Minimally Invasive Surgery, Beijing Chest Hospital, Capital Medical University; Beijing Tuberculosis and Thoracic Tumor Research Institute, Beijing, China.; 2 Department of Thoracic Surgery, National Cancer Center/National Clinical Research Center for Cancer, Cancer Hospital & Shenzhen Hospital, Chinese Academy of Medical Sciences and Peking Union Medical College, Shenzhen, China.; 3 State Key Laboratory of Oncology in South China, Department of Thoracic Surgery, Collaborative Innovation Center for Cancer Medicine, Sun Yat-sen University Cancer Center, Guangzhou, China.

**Keywords:** non-small cell lung cancer (NSCLC), propensity score matching (PSM), pulmonary ligament lymph nodes, overall survival (OS), recurrence-free survival (RFS)

## Abstract

**Objective:** The purpose of this study was to explore whether the resection of pulmonary ligament lymph nodes would affect the prognosis of patients with stage IB non-small cell lung cancer (NSCLC).

**Methods:** We retrospectively analyzed 341 patients with upper lobe stage IB NSCLC who underwent radical surgery for lung cancer at Sun Yat-Sen University Cancer Center from 1999 to 2009. The Cox proportional hazard regression model was used to analyze the prognostic factors. After propensity score matching (PSM), 204 cases were selected. The Kaplan-Meier method and log-rank test were applied to compare overall survival (OS) and recurrence-free survival (RFS).

**Results:** Among the 341 cases included in the study, 217 had no pulmonary ligament lymph nodes resected, and 124 had pulmonary ligament lymph nodes resected. They were divided into two groups according to whether the pulmonary ligament lymph nodes were resected; there were significant differences between the two groups in laterality, resected lymph node stations, and resected lymph node numbers (*P*<0.05). Univariate and multivariate analyses by the Cox proportional hazards model showed that age and family history of malignant tumors were prognostic factors for OS, and no variables were prognostic factors for RFS (*P*<0.05). Resection of the pulmonary ligament lymph node was not associated with OS or RFS. After propensity score matching (PSM), survival analysis was performed again using the Kaplan-Meier method and log-rank test; the results suggested that resection of the pulmonary ligament lymph node is not statistically associated with OS and RFS (*P*>0.05).

**Conclusions:** For stage IB NSCLC, resection of the pulmonary ligament lymph nodes was not statistically associated with OS or RFS. Pulmonary ligament lymph node resection is not necessary for early-stage NSCLC.

## Introduction

Non-small cell lung cancer (NSCLC), accounting for approximately 85% of lung cancers, is the most common pathological type of lung cancer 1. Surgery is currently the primary treatment option for NSCLC 2. For many years, lobectomy with systematic lymph node dissection (SLND) has been the standard surgical treatment for lung cancer 3, 4. However, for early-stage NSCLC, the method of lymph node dissection has been controversial. Many studies suggest that lobe-specific selective lymph node dissection (LSLND) and lymph node sampling (LNS) can be applied to early-stage NSCLC 5-11. These studies indicate that for early-stage NSCLC, LSLND and LNS can reduce operative time and some perioperative complications and have no difference in survival compared with SLND 6-9, 12. For tumors in the upper lobe, if SLND is performed, the pulmonary ligament lymph nodes need to be dissected. However, if LSLND or LNS is performed, pulmonary ligament lymph nodes do not need to be dissected. There are no studies on the necessity of dissection of pulmonary ligament lymph nodes (station 9 lymph nodes) in upper lobe NSCLC. The main purpose of this study was to explore the effect of pulmonary ligament lymph node dissection on survival in stage IB NSCLC.

## Methods

### Study cohort

We collected data from patients with NSCLC who underwent radical surgery at Sun Yat-Sen University Cancer Center from 1999 to 2009. The enrolled patients met the following requirements: 1) stage IB NSCLC located in the upper lobe; 2) treated with lobectomy; and 3) no history of neoadjuvant chemotherapy. Two experienced pathologists validated all pathological results. The exclusion criteria were as follows: 1) no detailed information on lymph nodes; 2) patients with autoimmune disease, preoperative infection, or severe cardiovascular disease; and 3) no detailed follow-up data. Ultimately, 341 cases were included in this study.

### Follow-up process

Follow-up was performed every 3 months for the first 2 years, every 6 months for 3 to 5 years, and once a year after that. Blood tests, tumor markers, chest CT, and abdominal ultrasound were performed to assess the patient's status. Once a year, a brain MRI is performed. All cases were followed up until January 2013.

### Statistical Analysis

To compare the variables between two groups, the chi-square test was used. Overall survival (OS) and recurrence-free survival (RFS) were calculated using the Kaplan-Meier method. The Cox proportional hazard regression model was performed for univariate and multivariate analyses. In multivariate analysis, variables with a *P* value less than 0.1 in univariate analysis were included. A *P* value of less than 0.05 was set as the statistical significance level.

R software (version 4.2.0, R Foundation, Austria) was used to perform the above statistical analyses. To conduct propensity score matching (PSM), the “matchit” package was used. Survival curves and forest plots were plotted using the “survival” and “survminer” packages. The optimal cutoff values for resected lymph node numbers were calculated using X-tile software (version 3.6.1, Yale University, United States).

## Results

### Patient characteristics

The clinicopathological characteristics of the patients in this study are shown in Table 1. A total of 341 patients were enrolled and divided into 2 groups according to whether the pulmonary ligament lymph nodes were resected. The chi-square test showed that there was a significant difference in laterality, resected lymph node stations, and resected lymph node numbers between the two groups (*P* < 0.05). We can see that male patients accounted for 72.7%. Patients older than 65 years accounted for approximately 31.4%. Patients with a history of smoking accounted for 58.7%. Most patients had no family history of malignant tumors (87.4%). Patients with grade I+II accounted for about 62.8%. The pathological type of the majority of patients was adenocarcinoma (67.7%). Squamous cell carcinoma accounted for approximately 29.9%, and the remaining included sarcomatoid carcinoma, mucoepidermoid carcinoma, and large cell carcinoma. Tumors located in the right lung accounted for 53.4%. Visceral pleural invasion and bronchial invasion accounted for 60.4% and 27.0%, respectively. A total of 28.4% of the patients had 6 or more lymph node dissection stations, and 59.5% had more than 12 lymph nodes dissected. Approximately 15.5% of patients received adjuvant chemotherapy after surgery.

### Prognostic factors for OS and RFS before PSM

**Figure 1:**
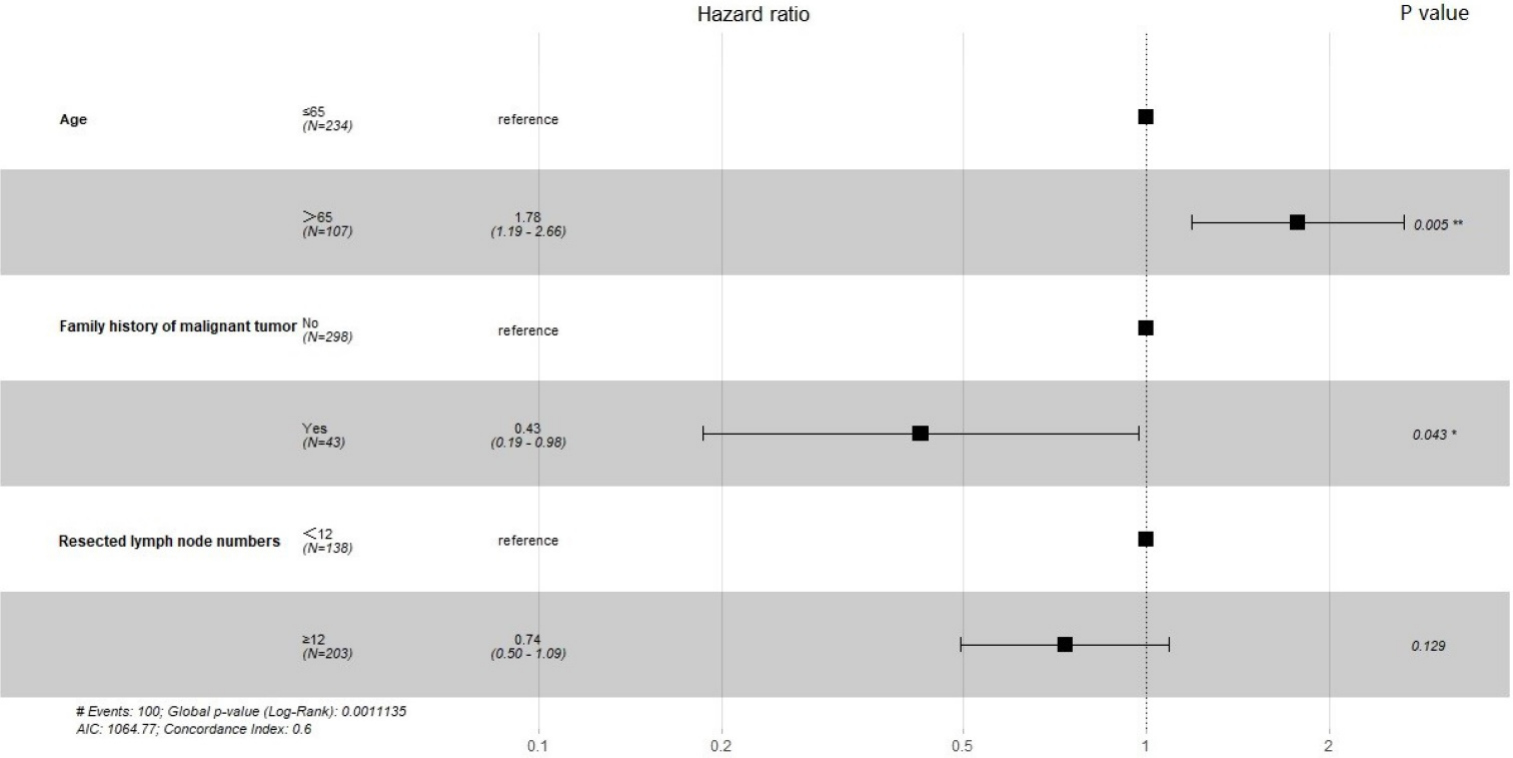
Forest plot showing multivariate analysis for Overall Survival.

Prognostic factors for OS and RFS were assessed using Cox proportional hazards regression models before PSM. The results of univariate analysis for OS and RFS are shown in Table 2. For OS, age, family history of malignant tumors, and resected lymph node numbers were included in multivariate analyses (*P*<0.1). For RFS, in univariate analysis, no variable had a P value less than 0.1, so all variables were considered to be not statistically associated with RFS. The forest plots revealed the results of the multivariate analysis. Figure 1 shows that the *P* values for age and family history of malignant tumors were less than 0.05. These variables were found to be significant prognostic factors for OS. Pulmonary ligament lymph node resection is not a prognostic factor for OS and RFS.

### Survival Analysis after PSM

**Figure 2:**
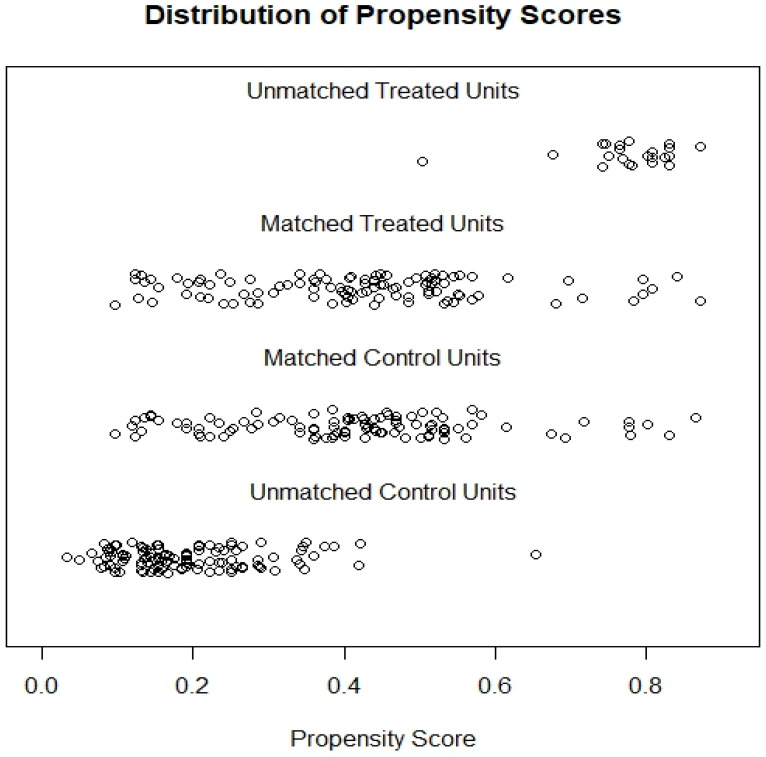
Distribution of propensity score.

To reduce potential bias in comparing the effects of pulmonary ligament lymph node resection on survival, a 1-to-1 PSM was performed. The distribution of propensity scores is shown in Figure 2; a perfect match is obtained between the 2 groups. A total of 204 cases were selected for survival analysis after PSM. As shown in Table 1, after PSM, there was no significant difference in any of the variables between the two groups (*P*>0.05).

**Figure 3:**
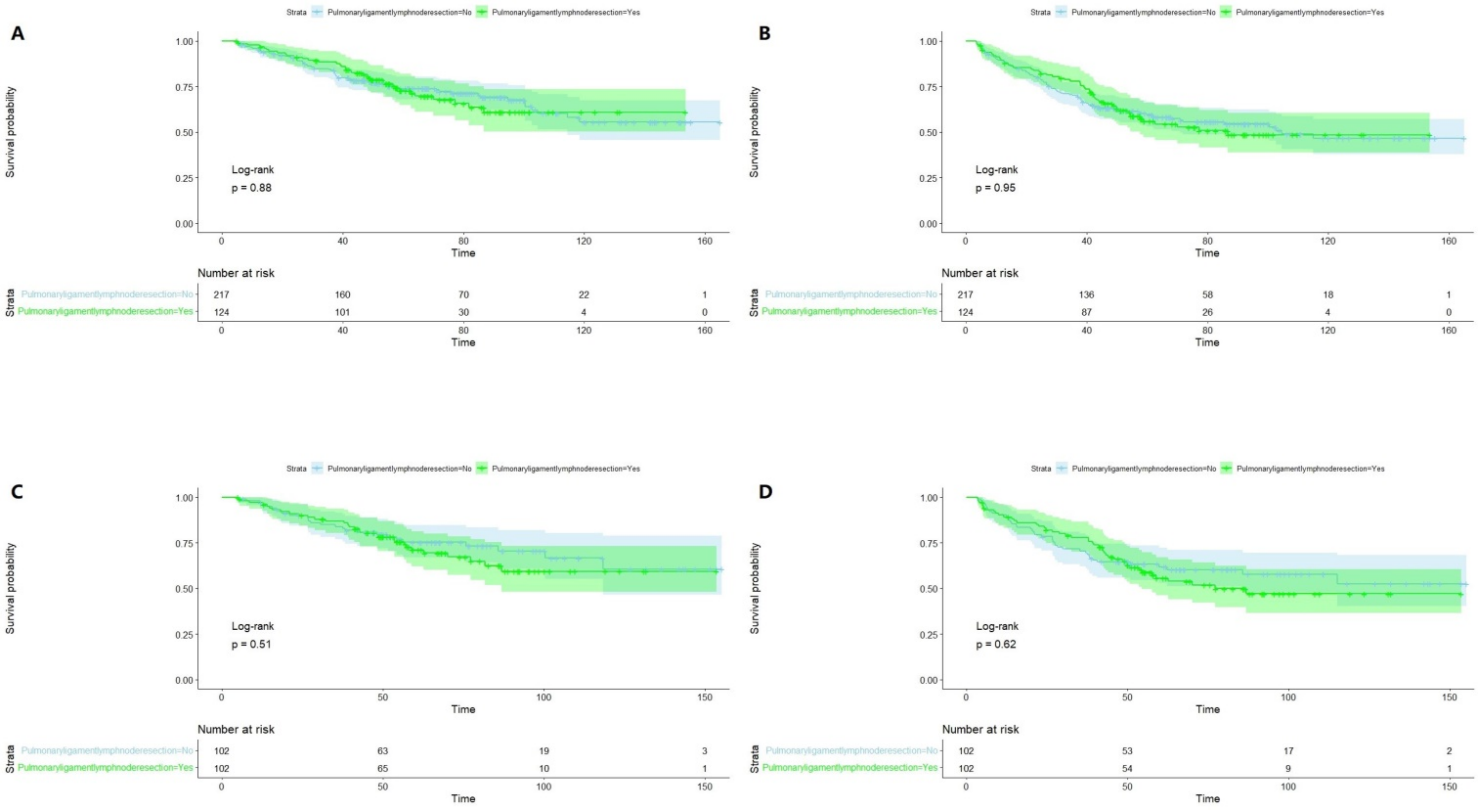
Kaplan-Meier curves of Overall Survival (**A, C**) and Recurrence-Free Survival (**B, D**) before and after propensity score matching.

Figure 3 illustrates Kaplan-Meier curves of OS (A, C) and RFS (B, D) before and after PSM. All P values were greater than 0.05, suggesting that pulmonary ligament lymph node resection was not statistically associated with OS or RFS.

## Discussion

Lymph node dissection is an integral part of lung cancer surgery, and the extent of lymph node dissection has always been a hot topic. SLND has been the standard surgical procedure for lung cancer surgery 13-15. However, there are different views on the extent of lymph node dissection in lung cancer. Especially for early-stage non-small cell lung cancer, several studies have concluded that SLND is not necessary. Many recent studies have suggested that LSLND and LNS can achieve the same therapeutic effect as SLND for early-stage NSCLC 5, 6, 9, 12, 16, 17. At the same time, some studies suggest that LSND and LNS can reduce the incidence of complications caused by lymph node dissection, such as lymphatic fistula, bleeding, and nerve damage 18. However, this view has different opinions 19-21.

Pulmonary ligament lymph nodes (station 9 lymph nodes) are located in the inferior pulmonary ligament, below the inferior pulmonary vein, and belong to the inferior mediastinal lymph nodes. In SLND, pulmonary ligament lymph nodes must be dissected. However, for LSLND and LNS, pulmonary ligament lymph nodes may not be dissected for upper lobe tumors 6, 8, 9, 17. In addition, some studies have concluded that tumors in the upper lobe are more likely to metastasize to the upper mediastinal lymph nodes 12, 22, 23, and the pulmonary ligament lymph nodes are less likely to metastasize than other mediastinal lymph nodes 24. Therefore, it is still controversial whether pulmonary ligament lymph nodes need to be dissected for early-stage upper lobe NSCLC. Since the dissection of the pulmonary ligament lymph nodes requires cutting the inferior pulmonary ligament, some studies have indicated that removing the inferior pulmonary ligament may lead to changes in the bronchial angle, resulting in bronchial obstruction and cough symptoms and shortness of breath 25, 26. At the same time, other studies confirmed that pulmonary ligament resection is not necessary for upper lobectomy 26, 27. This makes it more meaningful to study whether resection of pulmonary ligament lymph nodes is essential for early-stage upper lobe lung cancer.

This study retrospectively analyzed 341 patients with stage IB upper lobe NSCLC; prognostic analysis was performed using a Cox proportional hazard regression model before PSM. Univariate and multivariate analyses showed that pulmonary ligament lymph node resection was not a prognostic factor for OS or RFS. Subsequently, to reduce potential bias, all patients enrolled in the study were divided into two groups according to whether the pulmonary ligament lymph nodes were resected and matched with a 1:1 propensity score. Survival analysis was then performed by the Kaplan-Meier method, and the log-rank test indicated that the P values ​​were all greater than 0.05 (Figure 3), suggesting that pulmonary ligament lymph node resection was not statistically associated with OS and RFS. The conclusions of this study are consistent with those of previous studies on early-stage NSCLC 8, 10, 28. It should also be noted that for advanced NSCLC, SLND should be the standard surgical modality because of its survival benefit 29-31.

This study shows that the dissection of pulmonary ligament lymph nodes is not statistically associated with OS and RFS. For OS, only age and family history of malignant tumors were statistically significant prognostic factors. None of the variables included were prognostic factors for RFS. As shown in Figure 1, the hazard ratio of family history of malignant tumors was 0.43, with a *P* value less than 0.05. This means that patients with a family history of malignant tumors have a lower risk of death. This result may be related to the bias caused by the small sample size. We look forward to more studies with larger sample sizes to validate this conclusion.

Unavoidably, this study has several limitations. First, this is a single-center retrospective study, and prospective multicenter research is expected to confirm the conclusion. Second, 341 patient cases were enrolled in this study, and the small sample size may have led to bias. Finally, there are many surgical teams in our hospital, and the impact of the surgical procedures of the different surgical teams was not taken into account.

## Conclusion

For stage IB NSCLC, resection of the pulmonary ligament lymph nodes was not statistically associated with OS or RFS. Pulmonary ligament lymph node resection is not necessary for early-stage NSCLC.

## Figures and Tables

**Table 1:** Comparison of clinicopathological characteristics between original and matched data set

Variables	Original data set				Matched data set			
	Total	Pulmonary ligament lymph node resection		*P* value	Total	Pulmonary ligament lymph node resection		*P* value
	N=341	No (N=217)	Yes (N=124)		N=204	No (N=102)	Yes (N=102)	
**Sex**				0.151				0.762
Female	93 (27.3%)	53 (24.4%)	40 (32.3%)		63 (30.9%)	30 (29.4%)	33 (32.4%)	
Male	248 (72.7%)	164 (75.6%)	84 (67.7%)		141 (69.1%)	72 (70.6%)	69 (67.6%)	
**Age**				0.559				0.641
>65	107 (31.4%)	71 (32.7%)	36 (29.0%)		58 (28.4%)	27 (26.5%)	31 (30.4%)	
≤65	234 (68.6%)	146 (67.3%)	88 (71.0%)		146 (71.6%)	75 (73.5%)	71 (69.6%)	
**Smoking**				1.000				1.000
No	142 (41.6%)	90 (41.5%)	52 (41.9%)		88 (43.1%)	44 (43.1%)	44 (43.1%)	
Yes	199 (58.4%)	127 (58.5%)	72 (58.1%)		116 (56.9%)	58 (56.9%)	58 (56.9%)	
**Family history of malignant tumor**				0.770				1.000
No	298 (87.4%)	191 (88.0%)	107 (86.3%)		178 (87.3%)	89 (87.3%)	89 (87.3%)	
Yes	43 (12.6%)	26 (12.0%)	17 (13.7%)		26 (12.7%)	13 (12.7%)	13 (12.7%)	
**Grade**				0.695				0.662
I+II	214 (62.8%)	134 (61.8%)	80 (64.5%)		130 (63.7%)	63 (61.8%)	67 (65.7%)	
III+IV	127 (37.2%)	83 (38.2%)	44 (35.5%)		74 (36.3%)	39 (38.2%)	35 (34.3%)	
**Histology**				0.284				1.000
Adenocarcinoma	231 (67.7%)	149 (68.7%)	82 (66.1%)		137 (67.2%)	69 (67.6%)	68 (66.7%)	
Squamous cell carcinoma	102 (29.9%)	61 (28.1%)	41 (33.1%)		66 (32.4%)	33 (32.4%)	33 (32.4%)	
Others	8 (2.4%)	7 (3.2%)	1 (0.8%)		1 (0.4%)	0 (0.0%)	1 (0.9%)	
**Laterality**				<0.001				1.000
Left	159 (46.6%)	82 (37.8%)	77 (62.1%)		110 (53.9%)	55 (53.9%)	55 (53.9%)	
Right	182 (53.4%)	135 (62.2%)	47 (37.9%)		94 (46.1%)	47 (46.1%)	47 (46.1%)	
**Visceral pleura invasion**				0.746				0.771
No	135 (39.6%)	84 (38.7%)	51 (41.1%)		75 (36.8%)	36 (35.3%)	39 (38.2%)	
Yes	206 (60.4%)	133 (61.3%)	73 (58.9%)		129 (63.2%)	66 (64.7%)	63 (61.8%)	
**Bronchial invasion**				0.125				0.638
No	249 (73.0%)	165 (76.0%)	84 (67.7%)		148 (72.5%)	76 (74.5%)	72 (70.6%)	
Yes	92 (27.0%)	52 (24.0%)	40 (32.3%)		56 (27.5%)	26 (25.5%)	30 (29.4%)	
**Resected lymph node stations**				<0.001				1.000
<6	244 (71.6%)	178 (82.0%)	66 (53.2%)		130 (63.7%)	65 (63.7%)	65 (63.7%)	
≥6	97 (28.4%)	39 (18.0%)	58 (46.8%)		74 (36.3%)	37 (36.3%)	37 (36.3%)	
**Resected lymph node numbers**				0.002				0.883
<12	138 (40.5%)	102 (47.0%)	36 (29.0%)		70 (34.3%)	34 (33.3%)	36 (35.3%)	
≥12	203 (59.5%)	115 (53.0%)	88 (71.0%)		134 (65.7%)	68 (66.7%)	66 (64.7%)	
**Chemotherapy**				0.316				1.000
No	288 (84.5%)	187 (86.2%)	101 (81.5%)		166 (81.4%)	83 (81.4%)	83 (81.4%)	
Yes	53 (15.5%)	30 (13.8%)	23 (18.5%)		38 (18.6%)	19 (18.6%)	19 (18.6%)	

**Table 2:** Univariate analysis of overall survival and recurrence free survival before propensity score matching

Variables	Total	Overall survival		Recurrence free survival	
		HR (95% CI)	*P* value	HR (95% CI)	*P* value
**Sex**			0.733		0.547
Female	93 (27.3%)	reference		reference	
Male	248 (72.7%)	0.93 (0.59-1.44)		0.90 (0.63-1.28)	
**Age**			0.004		0.109
>65	107 (31.4%)	reference		reference	
≤65	234 (68.6%)	0.55 (0.37-0.83)		0.76 (0.54-1.06)	
**Smoking**			0.174		0.627
No	142 (41.6%)	reference		reference	
Yes	199 (58.4%)	1.32 (0.88-1.99)		1.08 (0.78-1.50)	
**Family history of malignant tumor**			0.023		0.273
No	298 (87.4%)	reference		reference	
Yes	43 (12.6%)	0.40 (0.17-0.90)		0.74 (0.44-1.27)	
**Grade**			0.181		0.254
I+II	214 (62.8%)	reference		reference	
III+IV	127 (37.2%)	1.31 (0.88-1.95)		1.21 (0.87-1.68)	
**Histology**			0.563		0.358
Adenocarcinoma	231 (67.7%)	reference		reference	
Squamous cell carcinoma	102 (29.9%)	1.10 (0.72-1.68)		0.81 (0.56-1.17)	
Others	8 (2.4%)	1.82 (0.57-5.80)		1.46 (0.54-3.97)	
**Laterality**			0.711		0.615
Left	159 (46.6%)	reference		reference	
Right	182 (53.4%)	0.93 (0.63-1.37)		1.09 (0.79-1.50)	
**Visceral pleura invasion**			0.216		0.516
No	135 (39.6%)	reference		reference	
Yes	206 (60.4%)	0.78 (0.53-1.16)		1.12 (0.80-1.56)	
**Bronchial invasion**			0.730		0.292
No	249 (73.0%)	reference		reference	
Yes	92 (27.0%)	0.92 (0.58-1.47)		0.81 (0.55-1.20)	
**Resected lymph node stations**			0.209		0.454
<6	244 (71.6%)	reference		reference	
≥6	97 (28.4%)	0.75 (0.47-1.18)		0.87 (0.61-1.25)	
**Resected lymph node numbers**			0.096		0.123
<12	138 (40.5%)	reference		reference	
≥12	203 (59.5%)	0.72 (0.48-1.06)		0.78 (0.56-1.07)	
**Pulmonary ligament lymph node resection**			0.881		0.947
No	217 (63.6%)	reference		reference	
Yes	124 (36.4%)	1.03 (0.68-1.56)		1.01 (0.72-1.41)	
**Chemotherapy**			0.173		0.630
No	288 (84.5%)	reference		reference	
Yes	53 (15.5%)	0.65 (0.35-1.21)		0.89 (0.57-1.41)	

## Abbreviations

NSCLC: non-small cell lung cancer
PSM: propensity score matching
SLND: systematic lymph nodes dissection
LSLND: lobe-specific selective lymph nodes dissection
LNS: lymph nodes sampling
OS: overall survival
RFS: recurrence-free survival
